# Cardiovascular Risk Factor Profiles and Disease in Black Compared to Other Africans with Chronic Kidney Disease

**DOI:** 10.1155/2021/8876363

**Published:** 2021-02-19

**Authors:** Hon-Chun Hsu, Chanel Robinson, Angela J. Woodiwiss, Gavin R. Norton, Patrick H. Dessein

**Affiliations:** ^1^Cardiovascular Pathophysiology and Genomics Research Unit, School of Physiology, Faculty of Health Sciences, University of the Witwatersrand, Johannesburg, South Africa; ^2^Nephrology Unit, Milpark Hospital, Johannesburg, South Africa; ^3^Internal Medicine Department, Faculty of Health Sciences, University of the Witwatersrand, Johannesburg, South Africa; ^4^Free University and University Hospital, Brussels, Belgium

## Abstract

**Methods:**

Cardiovascular risk factors, aortic and cardiac function, atherosclerosis extent, and cardiovascular event rates were assessed in 115 consecutive predialysis (*n* = 67) and dialysis patients (*n* = 48) including 46 black and 69 other (32 Asian, 28 white, and 9 mixed race) participants. Data were analysed in multivariable regression models.

**Results:**

Overall, black compared to other African CKD patients had less frequent carotid artery plaque (OR (95% CI) = 0.38 (0.16–0.91)) despite an increased cardiovascular risk factor burden. In receiver operator characteristic curve analysis, the Framingham score performed well in identifying non-black but not black CKD patients with carotid plaque (area under the curve (AUC) (95% CI) = 0.818 (0.714–0.921) and AUC (95% CI) = 0.556 (0.375–0.921), respectively). Black compared to other African predialysis patients experienced larger Framingham scores and more adverse nontraditional cardiovascular risk factors, impaired arterial and diastolic function but similar cardiovascular event rates (OR (95% CI) = 0.93 (0.22 to 3.87)). Among dialysis patients, black compared to other Africans had an overall similar traditional and nontraditional cardiovascular risk factor burden, similar arterial and diastolic function but increased systolic function (partial *R* = 0.356, *p* = 0.01 and partial *R* = 0.315, *p* = 0.03 for ejection fraction and stroke volume, respectively) and reduced cardiovascular event rates (OR (95% CI) = 0.22 (0.05 to 0.88)).

**Conclusion:**

Black compared to other African CKD patients have less frequent very high risk atherosclerosis and experience weaker cardiovascular risk factor-atherosclerotic CVD relationships. These disparities may be due to differences in epidemiological health transition stages. Among dialysis patients, black compared to other Africans have less cardiovascular events, which may represent a selection bias as previously documented in black Americans.

## 1. Introduction

The global prevalence of chronic kidney disease (CKD) was recently estimated at 9.1% [1], whereas that in sub-Saharan Africa was 10.7% and ranged from 6.6% to 14% in west compared to South African sites [2]. Most patients with CKD are more likely to die from cardiovascular disease (CVD) than develop kidney failure [3, 4]. CVD in CKD is mediated by adverse traditional cardiovascular risk factor profiles and renal disease-specific factors including calcium-phosphate imbalance, anaemia, chronic volume overload, and oxidative stress [3, 4]. The risk of cardiovascular disease increases from 1.5-fold in patients with stage 2 CKD to 20-fold in those with end-stage renal disease (ESRD) [3].

Presently available evidence on the effects of CKD on CVD originates in studies that were mostly performed in high income countries. Sub-Saharan Africa is a large continent that consists of low and middle income countries [5, 6]. The sub-Saharan African black population is currently undergoing rapid urbanization and, consequently, an epidemiological health transition [7]. Cardiovascular risk factor profiles and their impact on CVD as well as cardiovascular event phenotypes differ in low or middle compared to high-income populations [7]. Black South African persons currently experience smaller age-standardized mortality rates from ischemic heart disease than their non-black counterparts [8]. By contrast, age-standardized mortality rates due to cerebrovascular and hypertensive heart disease are much larger in black compared to other South Africans [8].

Compared to their white counterparts, black Americans with predialysis chronic kidney disease experience an enhanced risk of CVD mortality [9]. This disparity further increases with CKD severity [9]. However, survival is better in black compared to bhite Americans once they are on dialysis [9].

The extent to which CKD impacts CVD risk among black Africans is less well established. The respective evidence derives from investigations that included only patients on dialysis [10–13]. In two retrospective cohort studies, sepsis was a more frequent cause of death than CVD among predominantly black African dialysis patients [10, 12]. Another study revealed a markedly low prevalence of coronary and aortic calcification in black African dialysis patients [11]. Yet, Amira and colleagues [13] identified carotid artery plaque in 38.1% of 58 black and 26 non-black African dialysis patients. Notably, the mean age of participants in these studies was as low as 36 to 42 years [10–13]. In the present study, we compared cardiovascular risk factor profiles [14], large artery function including arterial stiffness, wave reflection and pressure pulsatility [14–16], left ventricular systolic and diastolic function [17], atherosclerosis extent, and cardiovascular event rates between black and other African predialysis and dialysis patients.

## 2. Patients and Methods

### 2.1. Patients

One hundred and fifteen consecutive predialysis or dialysis patients that included 46 black and 69 other (32 Asian, 28 white, and 9 mixed race) participants were recruited at the Milpark Hospital in Johannesburg, South Africa. Predialysis patients had a Chronic Kidney Disease Epidemiology Collaboration estimated glomerular filtration rate (eGFR) [18] of <60 ml/min.1.73 m^2^ upon enrolment. Patients with infection or/and active cancer were excluded. The study was approved by the University of the Witwatersrand Human (Medical) Research Ethics Committee (protocol no. M15-08-43) in Johannesburg, South Africa, and performed according to the 2013 revised Helsinki Declaration. Each patient gave written informed consent.

### 2.2. Methods

Baseline recorded characteristics comprised demographic features, CKD staging, lifestyle factors, and anthropometric parameters. Dialysis patients were investigated on the day before undergoing the respective procedure. Each of these patients was dialysed thrice weekly.

#### 2.2.1. Cardiovascular Risk Factors and Their Treatment

Traditional and nontraditional or kidney disease-related cardiovascular risk factors and their treatment were recorded using previously reported methods [14] and as given in the Supplementary Materials (methods) (available here). The overall major traditional cardiovascular risk factor burden was estimated by calculating the Framingham score [19]. For this study, a high phosphate concentration was identified in patients with a phosphate level of >1.42 mmol/l or/and when a phosphate lowering agent (calcium carbonate or sevelamer in 48 and 1 (black dialysis patient) cases, respectively) was used.

#### 2.2.2. Arterial Function

Central hemodynamic characteristics were determined using a high-fidelity SPC-301 micromanometer (Millar Instument, Inc., Houston, Texas), interfaced with a computer utilizing SphygmoCor software, version 9.0 (AtCor Medical Pty., Ltd., West Ryde, New South Wales, Australia). We evaluated arterial stiffness as estimated by aortic pulse wave velocity, wave reflection as represented by the augmentation index, reflected wave pressure and reflection magnitude, and pressure pulsatility measures including central systolic and pulse pressure, peripheral pulse pressure, pressure amplification, and forward wave pressure as previously reported [14] and given in the Supplementary Materials (methods).

#### 2.2.3. Left Ventricular Structure and Function

Echocardiography was performed as recommended by the American Society of Echocardiography convention [17] and using a Philips CX50 POC CompactXtreme Ultrasound System (Philips Medical Systems (Pty.) Ltd., USA) equipped with a 1.8–4.2 MHz probe that allowed for M-mode, 2-D, pulsed, and tissue Doppler measurements. We assessed left ventricular structure as represented by mass and hypertrophy, systolic function as estimated by ejection fraction and systolic volume, and diastolic function parameters comprising the early (*E*)/late (atrial) diastolic wave (*A*) ratio, the peak mitral annulus motion during early diastole (*e*′), and *E*/*e*′ ratio, as previously described [17] and given in the Supplementary Materials (methods).

#### 2.2.4. Carotid Atherosclerosis

Carotid artery ultrasound was performed using a Philips CX50 POC CompactXtreme Ultrasound System (Philips Medical Systems (Pty.) Ltd., USA) attached to a linear array 4.0–12.0 MHz probe. The software provided for semiautomated border detection gives markedly reproducible data as previously described [14]. Images of at least 1 cm length of the distal common carotid arteries were obtained. The optimal angle of incidence was used, defined as the longitudinal angle of approach where both branches of the internal and external carotid artery were visualized simultaneously. The carotid intima-media thickness (c-IMT) was defined as the mean of the left and right common carotid artery thickness. Plaque in the extracranial carotid tree was defined according to the Mannheim consensus criteria [20]. Carotid ultrasound measurements were made by the same observer that performed the arterial function and echocardiographic evaluations (CR). The intraobserver variability of ultrasound measurements is low in our setting [14].

#### 2.2.5. Cardiovascular Event Rates

Cardiovascular event rates included ischemic heart disease (acute myocardial infarction, percutaneous transluminal coronary angioplasty, and/or coronary artery bypass surgery), heart failure, and/or cerebrovascular and/or peripheral arterial disease that were confirmed by a cardiologist, neurologist, and vascular surgeon, respectively.

#### 2.2.6. Data Analysis

Data were analysed using the IBM SPSS statistics program (version 23.0 IBM, USA) and significance was set at *p* ≤ 0.05. Significance was consistently analysed with two-sided tests. Results are expressed as mean (SD) or median (interquartile range (IQR)) for continuous variables and percentages for categorical variables. Nonnormally distributed characteristics were logarithmically transformed before entering them in multivariable regression models.

We compared traditional and nontraditional cardiovascular risk factor profiles, arterial function parameters, left ventricular structure and function variables, atherosclerosis markers, and cardiovascular event rates between black and other African chronic kidney disease patients in age and sex adjusted multivariable regression models. Other established potential confounders or mediators of arterial function [14] were consistently adjusted for in additional models. We subsequently performed sensitivity analyses among predialysis as well as dialysis patients. Differences among the 4 investigated groups that included black and other African predialysis and black and other African dialysis patients were assessed by ANOVA, Kruskal–Wallis, and chi-square test for continuous normally distributed, continuous nonnormally distributed, and categorical variables, respectively. The performance of the Framingham score in identifying black and other African chronic kidney disease patients with very high CVD risk as represented by carotid plaque presence was determined in receiver operator characteristic (ROC) curve analysis.

## 3. Results

### 3.1. Baseline Patient Characteristics

As given in Table 1, mean age was 5.6 years smaller in black compared to other CKD African patients (*p* = 0.03). Sex and lifestyle factors did not differ in black compared to other African participants. In age and sex adjusted analysis, black patients were more frequently on dialysis (OR (95% CI) = 3.18 (1.41 to 7.08)). Body weight and height were each smaller in black compared to other African patients. These differences reached significance for weight (*p* = 0.02) only. Other anthropometric measures were similar in the two groups.

### 3.2. Traditional and Nontraditional Cardiovascular Risk Factors in Black Compared to Other African Patients with CKD

Cardiovascular risk factor profiles are presented in Table 2. In age and sex adjusted analysis, hypertension and diabetes were more prevalent in black compared to other African CKD patients. The use of insulin, diuretics, and calcium channel blockers was more frequent, whereas that of lipid lowering agents was less prevalent in black compared to other African patients. Systolic blood pressure, heart rate, and Framingham score were each larger in black compared to other African patients.

With regard to nontraditional cardiovascular risk factors, black patients had more frequently high phosphate concentrations, smaller calcium and haemoglobin levels, and larger parathyroid and ferritin concentrations compared to other African participants. Black patients also more often received erythropoietin stimulating agent and intravenous iron therapy. In an additional logistic regression model in which diuretic agent use was adjusted for, black population origin remained associated with low calcium levels (partial *R* = −0.192, *p* = 0.04). Black patients used erythropoietin stimulating agents and intravenous iron more often than their other African counterparts.

### 3.3. Arterial Function in Black Compared to Other African Patients with CKD

As given in Table 3, in age and sex adjusted analysis, pressure pulsatility as represented by central systolic and pulse pressure, peripheral pulse pressure, and forward wave pressure were each larger in black compared to other African CKD patients (model 1 in Table 3). Upon additional adjustment for other established confounders or mediators, these disparities persisted except for as related to the forward wave pressure.

### 3.4. Left Ventricular Structure and Function in Black Compared to Other African Patients with Chronic Kidney Disease

Cardiac structure and function measures in black and other African CKD patients are shown in Table 4. In age and sex adjusted analysis, black African patients experienced a larger *E*/*e*′ ratio and smaller *e*′.

### 3.5. Atherosclerosis and Cardiovascular Event Rates in Black Compared to Other African Patients with Chronic Kidney Disease

As given in Table 5, in age and sex adjusted analysis, carotid artery plaque prevalence was smaller in black compared to other African CKD patients. The frequency of cardiovascular events and carotid intima-media thickness was also smaller in black compared to other African patients, but none of these differences reached significance.

### 3.6. Sensitivity Analyses

#### 3.6.1. Baseline Characteristics in Black Compared to Other African Predialysis and Dialysis Patients

Baseline demographic characteristics, lifestyle factors, and anthropometric features did not differ significantly in black compared to other predialysis as well as dialysis patients, as shown in Table 1.

#### 3.6.2. Traditional and Nontraditional Cardiovascular Risk Factors in Black Compared to Other African Predialysis and Dialysis Patients

Table 2 gives the traditional and nontraditional cardiovascular risk factors in black compared to other African predialysis and dialysis patients. Among predialysis patients, all black African patients were hypertensive as compared to 81.3% of their other African counterparts. Black patients also had a larger systolic blood pressure and used diuretics, calcium channel blockers, and alpha blockers more frequently. Diabetes was more prevalent, haemoglobin A1C concentrations were larger, and insulin was used more frequently in black compared to other African patients. The Framingham score was larger in black compared to other African patients. With regard to nontraditional cardiovascular risk factors, the prevalence of high phosphate levels and parathyroid concentrations was larger and vitamin D levels were smaller in black compared to other African patients.

Among dialysis patients, beta blockers were used less frequently and heart rate was larger in black compared to other African patients. With regard to nontraditional cardiovascular risk factors, the calcium *x* phosphate product was smaller in black compared to other African patients.

#### 3.6.3. Arterial Function in Black Compared to Other African Predialysis and Dialysis Patients

Arterial function in black compared to other African predialysis and dialysis patients are shown in Table 3. Among predialysis patients, arterial wave reflection markers and, except for the forward wave pressure, pressure pulsatility parameters were larger in black compared to other African patients. Except for as related to augmentation index and reflection magnitude, each of these disparities remained significant in regression models in which, besides age and sex, other established potential confounders or mediators were additionally adjusted for.

Among dialysis patients, arterial stiffness and wave reflection as well as pressure pulsatility measures did not differ significantly in black compared to other African patients.

#### 3.6.4. Left Ventricular Structure and Function in Black Compared to Other African Predialysis and Dialysis Patients

Table 4 gives the left ventricular structure and function in black compared to other African predialysis and dialysis patients. Among predialysis patients, the *E*/*e*′ ratio was larger and the *e*′ was smaller in black compared to other African patients. The association of black population origin with *E*/*e*′ ratio was unaltered upon additional adjustment for left ventricular hypertrophy (partial *R* = 0.307, *p* = 0.01) or hypertension (partial *R* = 0.278, *p* = 0.02) but was no longer present after diabetes was adjusted for (partial *R* = 0.089, *p* = 0.5).

Among dialysis patients, ejection fraction and stroke volume were larger in black compared to other African patients. These associations were materially unaltered upon additional adjustment for haemoglobin levels (partial *R* = 0.356, *p* = 0.01 and partial *R* = 0.276, *p* = 0.07).

#### 3.6.5. Carotid Atherosclerosis and Cardiovascular Event Rates in Black Compared to Other African Predialysis and Dialysis Patients

Among predialysis patients, carotid atherosclerosis extent and cardiovascular event rates did not differ significantly in black compared to other African patients, as shown in Table 5.

Among dialysis patients, black compared to other African study participants were less likely to have experienced any cardiovascular event.

#### 3.6.6. Differences among Black and Other African Predialysis and Black and Other African Dialysis Patients

Differences among the 4 investigated groups that included black and other African predialysis and black and other African dialysis patients were confirmed upon intergroup comparisons. This was the case for body weight as a baseline characteristic (Table 1), a substantial proportion of the traditional and nontraditional cardiovascular risk factors and their treatment (Table 2), pressure pulsatility measures (Table 3), *E*/*e*′ (Table 4), and carotid artery plaque and intima-media thickness as well as ischemic heart disease (Table 5).

### 3.7. Association of Cardiovascular Risk Factors with Atherosclerosis in Black Compared with Other African Chronic Kidney Disease

**Figure 1:**
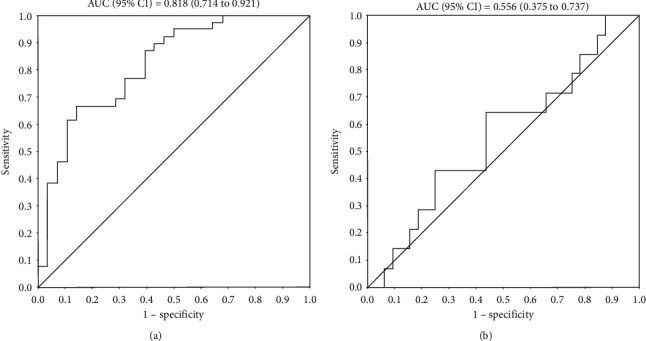
Performance of the Framingham score in identifying non-black (a) and black (b) CKD patients with carotid artery plaque.

The abovementioned results indicate that the cardiovascular disease risk factor burden was larger in black compared to other African chronic kidney disease patients. Despite this disparity, the atherosclerosis extent as estimated by carotid plaque prevalence was smaller in black compared to other African patients. In this regard, in interaction analysis, black population origin impacted the Framingham score-plaque prevalence relationship (OR (95% CI) = 0.914 (0.862 to 0.970), interaction *p* = 0.003). As given in Table 6, stratified analysis revealed that traditional cardiovascular risk factors were not related to carotid plaque in black African patients, whereas age, sex, dyslipidemia, and Framingham score were associated with atherosclerosis among other Africans. The performance of the Framingham score in identifying black and other African chronic kidney disease patients with carotid plaque in ROC curve analysis is shown in Figure 1. The area under the curve (AUC) (95% CI) for the association of the Framingham score with plaque was 0.556 (0.375 to 0.737) (*p* = 0.6) in black compared to 0.818 (0.714 to 0.921) (*p* < 0.0001) in other Africans.

Nontraditional cardiovascular risk factors were not associated with carotid plaque (data not shown).

## 4. Discussion

To our knowledge, this is the first study that compared the traditional and nontraditional cardiovascular risk factor burden and subclinical and established CVD between black and other predialysis and dialysis African patients that were seen at the same centre. The main novel findings produced by our investigation were as follows: (1) overall, black compared to other African CKD patients experienced a larger traditional cardiovascular risk factor burden as estimated by the Framingham score, more adverse nontraditional cardiovascular risk factors, and impaired arterial and diastolic function but less frequent very high risk atherosclerosis as represented by carotid artery plaque presence and numerically though not significantly smaller cardiovascular event rates; (2) black compared to other African predialysis patients experienced a larger traditional cardiovascular risk factor burden, more adverse nontraditional cardiovascular risk factors, impaired arterial function, and diastolic dysfunction but similar cardiovascular event rates; (3) among dialysis patients, black compared to other Africans had an overall similar traditional and nontraditional cardiovascular risk factor burden, similar arterial and diastolic function but increased systolic function, and reduced cardiovascular event rates; and (4) in ROC curve analysis among all participants, the Framingham score was strongly associated with carotid artery plaque in non-black but not black African CKD patients.

We found that, as applies to American black predialysis CKD patients [9], the prevalence of hypertension and diabetes was larger in black compared to other African study participants (100% versus 81.3% and 68.4% versus 16.7%, respectively). These differences translated into an overall increased major traditional cardiovascular risk factor burden. Moreover, hypertension was more severe in black predialysis CKD patients in that despite the use of more intensive antihypertensive therapy, their systolic blood pressure was larger than in their non-black counterparts. Additionally, black African CKD patients had more frequent high phosphate levels, larger parathyroid hormone concentrations, and lower vitamin D levels.

Severe arteriosclerosis is a characteristic vascular feature of CKD that results in impaired large artery function [21]. Increased arterial stiffness, wave reflection, and pressure pulsatility in black persons were well documented in general population studies [22–24]. These central artery characteristics contribute to heart failure, arrhythmias, sudden death, stroke, and myocardial infarction in CKD [15, 16]. In this study, black African predialysis CKD patients experienced increased wave reflection and pressure pulsatility. Increased wave reflection contributes to enhanced pressure pulsatility, which is strongly associated with CVD and disease progression in CKD patients [21, 25–27]. Effective management of impaired central artery function associates with improved survival in CKD [21]. Our results therefore argue for comprehensive cardiovascular risk factor management among black African predialysis CKD patients.

We measured *E*/*A* ratio and *e*′ as markers of left ventricular relaxation and *E*/*e*′ ratio as an index of left ventricular filling pressure. The *E*/*e*′ ratio and *e*′ predict incident cardiac and cardiovascular events more strongly than the *E*/*A* ratio [28]. In this study, the *E*/*e*′ ratio was larger and the *e*′ smaller in black compared to other African predialysis CKD patients. Hypertension and diabetes are both important determinants of diastolic dysfunction in the general population [29]. In multivariate analysis, we found that diabetes but not hypertension explained the association of black population origin with impaired diastolic function among predialysis CKD patients.

In contrast to black African predialysis patients, those on dialysis experienced similar cardiovascular risk factor profiles and arterial and left ventricular diastolic function to those recorded in other Africans. In this regard, Bellasi and colleagues [30] previously reported disparities in cardiovascular risk factors but similar arterial function in black compared to white American dialysis patients. More noticeably, we found that cardiovascular event rates were significantly reduced by 78% (OR (95% CI) = 0.22 (0.05 to 0.88)) in black compared to other African dialysis patients. This is remarkably similar to what was reported among black American dialysis patients [9, 30].The likelihood of progressing from chronic kidney disease to end-stage renal disease is greater in African Americans than whites. However, once on dialysis, survival is better among African Americans compared with whites. In 2008, the mortality rate for patients on dialysis was 16% in African Americans compared with 24% in whites. This disparity may be due to black predialysis CKD patients being sicker and therefore more likely to die, a phenomenon that would be expected to result in a selection of healthier persons that survive by the time they start dialysis [30]. This notion is supported by the DOPPS-1 report in which comorbidities, concurrent therapies, and nutritional variables explained the reduced mortality rates in black compared to white dialysis patients [31]. The authors concluded that minority group dialysis patients should not be expected to survive longer than their white counterparts with similar characteristics. Interestingly, we also found that left ventricular function as estimated by the ejection fraction and stroke volume was more preserved in black compared to other African dialysis patients.

Besides the decreased cardiovascular event rates in black compared to other African dialysis patients, the most striking finding in this study was that, among all study participants, despite the more adverse cardiovascular risk factor profiles, impaired arterial function, and left ventricular diastolic dysfunction, the atherosclerosis extent as represented by plaque presence was overall smaller in black compared to other African CKD patients (OR (95% CI) = 0.38 (0.16 to 0.91)). Additionally, in black compared to other African predialysis patients, despite more adverse cardiovascular risk factor profiles, cardiovascular event rates were not increased (OR (95% CI) = 0.93 (0.0.22 to 3.87)). These results suggest that cardiovascular risk factors may be less strongly associated with atherosclerotic CVD in black compared to other African CKD patients. Indeed, black population origin impacted the Framingham score-plaque prevalence relationship (interaction *p* = 0.003). In stratified analysis, the Framingham score was associated with carotid plaque in non-black but not black African CKD patients. ROC curve analysis confirmed that the Framingham score performed well in identifying very high risk atherosclerosis in non-black but not black African CKD patients (AUC (95% CI) = 0.818 (0.714 to 0.921) and AUC (95% CI) = 0.556 (0.375 to 0.921)), respectively. We recently reported the same finding in black African patients with rheumatoid arthritis [32–34]. This may be attributable to a shorter lifetime exposure to cardiovascular risk factors as part of recent urbanization and an earlier epidemiological transition stage [32]. More importantly, this finding indicates that cardiovascular risk equations such as the Framingham score should not be relied upon in cardiovascular risk stratification among black African CKD patients [33, 34].

The major limitations of the present study are the relatively small number of patients included, particularly in subgroups, its cross-sectional design, and that all included participants were enrolled at a single centre. Strengths are that we performed a detailed assessment of not only cardiovascular risk factor profiles but also large artery and cardiac function as well as subclinical atherosclerosis and cardiovascular events, and that recorded data were compared between black and other African CKD patients.

## 5. Conclusion

Overall, black compared to other African CKD patients currently experience less frequent severe atherosclerosis despite an increased cardiovascular risk factor burden. The Framingham score is useful in atherosclerotic CVD risk stratification among non-black but not black African CKD patients. Among predialysis patients, black compared to other Africans have more adverse traditional and nontraditional cardiovascular risk factor profiles, impaired arterial function, and diastolic dysfunction but similar cardiovascular event rates. These disparities may originate in differences in epidemiological transition stages. Black compared to other African dialysis patients have smaller cardiovascular event rates, which may represent a selection bias as previously documented in black Americans.

## Figures and Tables

**Table 1:** Baseline characteristics in black compared to other African chronic kidney disease patients overall and in sensitivity analysis among predialysis and dialysis patients.

Characteristics	Chronic kidney disease patients									
	All patients			Predialysis patients			Dialysis patients			Intergroup comparison^a^
	Black African (*n* = 46)	Other African (*n* = 69)	*p* value	Black African (*n* = 19)	Other African (*n* = 48)	*p* value	Black African (*n* = 27)	Other African (*n* = 21)	*p* value	*p* value
*Demographics*										
Age (years)	**54.3 (14.6)**	**59.9 (13.3)**	**0.03**	55.7 (14.2)	60.3 (13.6)	0.2	53.3 (15.0)	59.1 (13.0)	0.2	0.1
Female sex	18 (39.1)	25 (36.2)	1.0	6 (31.6)	15 (31.3)	0.8	12 (44.4)	10 (47.6)	0.8	0.5

*CKD stage*										
Predialysis	**19 (41.3)**	**48 (69.6)**	**0.005**	—	—	—	—	—	—	—
Dialysis	**27 (58.7)**	**21 (30.4)**	**0.005**	—	—	—	—	—	—	—

*Lifestyle*										
Current smoker	2 (4.4)	1 (1.5)	0.4	2 (10.5)	1 (2.1)	0.2	0 (0)	0 (0)	—	—
Exsmoker	2 (4.4)	6 (8.7)	0.3	0 (0)	4 (8.3)	1.0	2 (7.4)	2 (9.5)	0.5	0.6
Alcohol	1 (2.2)	1 (1.5)	0.8	0 (0)	1 (2.1)	1.0	1 (3.7)	0 (0)	—	0.7
Exercise	19 (41.3)	23 (33.3)	0.4	6 (31.6)	11 (22.9)	0.5	13 (48.2)	12 (57.1)	0.4	0.06

*Anthropometry*										
Weight (kg)	**74.5 (13.0)**	**81.1 (16.8)**	**0.02**	76.3 (13.6)	83.1 (16.5)	0.07	73.1 (12.7)	76.7 (17.1)	0.5	**0.04**
Height (cm)	167.8 (9.8)	170.5 (10.4)	0.09	170.0 (7.9)	171.3 (9.7)	0.5	166.2 (10.7)	168.6 (11.3)	0.2	0.2
Waist (cm)	96.5 (13.1)	102.0 (15.3)	0.1	97.1 (10.4)	101.4 (14.8)	0.3	96.1 (14.9)	103.3 (16.7)	0.3	0.2
Neck (cm)	38.6 (3.8)	39.7 (4.5)	0.2	38.7 (4.7)	39.5 (5.5)	0.7	38.4 (3.1)	40.1 (5.1)	0.3	0.5
BMI (kg/m^2^)	26.6 (5.3)	27.9 (5.6)	0.2	26.5 (5.2)	28.3 (5.5)	0.2	26.7 (5.5)	27.0 (5.8)	0.8	0.4
Waist-hip ratio	0.98 (0.11)	0.97 (0.10)	0.2	0.97 (0.14)	0.97 (0.11)	0.6	0.99 (0.09)	0.97 (0.09)	0.3	0.8
Waist-height ratio	0.58 (0.09)	0.59 (0.09)	0.5	0.57 (0.07)	0.59 (0.09)	0.5	0.58 (0.11)	0.61 (0.11)	0.7	0.5

Data are expressed as mean (SD) or number (percent) and were analysed in age and sex adjusted linear of logistic regression models as appropriate. ^a^For differences among black and other African predialysis patients and black and other African dialysis patients. Significant differences are shown in bold. CKD, chronic kidney disease; BMI, body mass index.

**Table 2:** Traditional and nontraditional cardiovascular risk factors and their treatment in black compared to other African chronic kidney disease patients overall and in sensitivity analysis among predialysis and dialysis patients.

Characteristics	Chronic kidney disease patients												
	All patients				Predialysis patients				Dialysis patients				Intergroup comparison^b^
	Black African (*n* = 46)	Other African (*n* = 69)	Model^a^		Black African (*n* = 19)	Other African (*n* = 48)	Model^a^		Black African (*n* = 27)	Other African (*n* = 21)	Model^a^		*p* value
*Traditional CV risk factors and their treatment*													
Categorical variables			OR (95% CI)				OR (95% CI)				OR (95% CI)		
Hypertension	**45 (97.8)**	**59 (85.5)**	**9.05 (1.08–75.59)**		19 (100)	39 (81.3)	—		26 (96.3)	20 (95.2)	1.51 (0.08–28.03)		**0.04**
Dyslipidemia	30 (71.4)	53 (85.5)	0.41 (0.15–1.12)		12 (70.6)	41 (91.1)	0.25 (0.06–1.10)		18 (72.0)	12 (70.6)	0.89 (0.21–3.72)		0.1
Diabetes	**21 (45.7)**	**19 (27.5)**	**3.10 (1.30–7.40)**		**13 (68.4)**	**8 (16.7)**	**13.28 (5.51–50.27)**		8 (29.6)	11 (52.4)	0.51 (0.13–2.01)		**<0.0001**
Antihypertensive treatment	**45 (97.8)**	**59 (85.5)**	**9.05 (1.08–75.59)**		19 (100)	39 (81.3)	—		26 (96.3)	20 (95.2)	1.51 (0.08–28.03)		**0.04**
ACEI/ARB	36 (81.8)	54 (79.4)	1.07 (0.40–2.87)		15 (83.3)	38 (79.2)	1.41 (0.33–6.03)		21 (80.1)	16 (80.0)	0.70 (0.14–3.47)		1.0
Beta blocker	21 (47.7)	30 (44.4)	1.22 (0.51–2.45)		9 (50.0)	16 (33.3)	2.31 (0.72–7.26)		**12 (46.2)**	**14 (70.0)**	**0.20 (0.05–0.88)**		**0.04**
Diuretic	**20 (44.4)**	**19 (27.5)**	**2.56 (1.11–5.94)**		**11 (61.1)**	**13 (27.1)**	**5.52 (1.59–19.17)**		9 (33.3)	6 (28.6)	1.34 (0.36–5.05)		0.07
Calcium channel blocker	**26 (57.8)**	**23 (33.8)**	**2.70 (1.22–6.00)**		**10 (52.6)**	**11 (22.9)**	**3.69 (1.18–11.50)**		16 (61.5)	12 (60.0)	1.14 (0.33–3.91)		**0.002**
Alpha blocker	11 (25.6)	13 (19.1)	1.73 (0.67–4.48)		**7 (38.9)**	**8 (16.7)**	**5.48 (1.31–22.87)**		4 (16.0)	5 (25.0)	0.53 (0.11–2.45)		0.2
Lipid lowering therapy	**21 (47.7)**	**51 (75.0)**	**0.33 (0.14–0.75)**		9 (50.0)	37 (77.1)	0.32 (0.09–1.08)		12 (46.2)	14 (70.0)	0.31 (0.08–1.12)		**0.03**
Statin	**21 (47.7)**	**51 (75.0)**	**0.33 (0.14–0.75)**		9 (50.0)	37 (77.1)	0.32 (0.09–1.08)		12 (46.2)	14 (70.0)	0.31 (0.08–1.12)		**0.03**
Ezetimibe	4 (9.1)	7 (10.4)	0.96 (0.26–3.61)		2 (11.1)	5 (10.4)	1.26 (0.21–7.59)		2 (7.7)	2 (10.5)	0.72 (0.09–5.95)		1.0
Insulin	**14 (31.1)**	**12 (17.4)**	**2.60 (1.03–6.61)**		**9 (47.4)**	**5 (10.4)**	**7.67 (2.07–28.41)**		5 (19.2)	7 (33.3)	0.67 (0.16–2.89)		**0.007**
OGLA	8 (17.8)	6 (8.7)	3.37 (0.98–11.61)		4 (21.1)	4 (8.3)	3.26 (0.64–16.56)		4 (15.4)	2 (9.5)	3.24 (0.40–26.49)		0.5
Continuous variables			Partial *R*	*p*			Partial *R*	*p*			Partial *R*	*p*	
Systolic blood pressure	**148 (24)**	**137 (18)**	**0.271**	**0.004**	**145 (25)**	**135 (16)**	**0.265**	**0.03**	150 (24)	141 (21)	0.185	0.2	**0.02**
Diastolic blood pressure (mmHg)	84 (13)	82 (10)	0.032	0.7	80 (10)	82 (8)	−0.150	0.2	86 (13)	82 (15)	0.083	0.6	0.2
Mean blood pressure (mmHg)	105 (14)	100 (11)	0.175	0.06	101 (13)	100 (9)	0.093	0.5	107 (14)	102 (14)	0.155	0.3	0.07
Total cholesterol (mmol/l)	4.3 (1.2)	4.2 (1.2)	0.005	1.0	4.2 (1.2)	4.2 (1.1)	−0.006	1.0	4.3 (1.2)	4.3 (1.3)	−0.001	1.0	1.0
LDL cholesterol (mmol/l)	2.3 (0.9)	2.4 (1.0)	−0.047	0.6	2.2 (0.7)	2.3 (0.9)	−0.094	0.5	2.4 (1.0)	2.5 (1.1)	−0.038	0.8	0.8
HDL cholesterol (mmol/l)	1.14 (0.43)	1.14 (0.42)	−0.007	0.9	1.03 (0.37)	1.12 (0.42)	−0.087	0.5	1.22 (0.45)	1.18 (0.41)	0.058	0.7	0.5
Non-HDL cholesterol (mmol/l)	3.1 (1.1)	3.1 (1.0)	−0.017	0.8	3.2 (1.3)	3.1 (1.0)	0.025	0.9	3.1 (1.1)	3.3 (1.1)	−0.086	0.6	0.9
Triglycerides (mmol/l)	1.3 (0.9–1.8)	1.5 (1.1–2.1)	−0.080	0.4	1.5 (1.1–1.9)	1.4 (1.1–2.2)	0.033	0.8	1.4 (0.8–1.8)	1.6 (1.2–1.8)	−0.127	0.4	0.4
Cholesterol-HDL cholesterol ratio	3.8 (2.7–5.2)	3.8 (3.1–4.8)	−0.022	0.6	3.7 (3.1–5.4)	3.8 (3.1–4.9)	0.073	0.6	3.8 (2.6–5.1)	3.9 (3.1–4.8)	−0.122	0.4	0.9
Triglycerides-HDL cholesterol ratio	1.2 (0.7–2.0)	1.4 (0.9–2.1)	−0.050	0.8	1.7 (1.2–2.3)	1.5 (1.0–2.1)	0.102	0.4	1.0 (0.6–2.0)	1.3 (0.9–2.4)	−0.111	0.5	0.2
Haemoglobin A1C (%)	5.6 (5.1–7.0)	5.5 (5.2–6.3)	0.106	0.3	**6.9 (5.7–8.3)**	**5.6 (5.3–6.2)**	**0.429**	**<0.0001**	5.4 (4.9–5.7)	5.4 (4.9–7.3)	−0.094	0.5	**0.004**
Framingham score	**23.4 (21.7)**	**22.6 (16.5)**	**0.223**	**0.01**	**27.9 (23.3)**	**21.8 (16.3)**	**0.327**	**0.008**	20.2 (20.2)	24.3 (14.0)	0.046	0.8	0.5
Heart rate (beats per minute)	**79 (14)**	**72 (14)**	**0.196**	**0.04**	76 (15)	72 (15)	0.067	0.6	**81 (13)**	**72 (12)**	**0.350**	**0.02**	**0.05**
Antihypertensives (*n*)	**2.5 (1.1)**	**2.0 (1.2)**	**0.215**	**0.02**	**2.8 (1.2)**	**2.0 (1.2)**	**0.388**	**0.001**	2.3 (1.1)	2.5 (1.1)	−0.149	0.3	**0.008**

*Nontraditional CV risk factors and their treatment*													
Categorical variables			OR (95% CI)				OR (95% CI)				OR (95% CI)		
High phosphate	**34 (77.3)**	**31 (44.9)**	**3.99 (1.67–9.54)**		**9 (52.9)**	**12 (25.0)**	**3.31 (1.01–10.89)**		25 (92.6)	19 (90.5)	1.20 (0.15–9.92)		**<0.0001**
Phosphate binder	22 (52.4)	25 (37.3)	1.71 (0.77–3.81)		4 (23.5)	8 (16.7)	1.55 (0.39–6.10)		18 (72.0)	17 (89.5)	0.27 (0.05–1.52)		**<0.0001**
Vitamin D replacement	27 (67.5)	31 (47.7)	2.27 (0.98–5.28)		9 (50.0)	14 (31.1)	1.96 (0.62–6.17)		18 (81.8)	17 (85.0)	1.06 (0.19–6.03)		**<0.0001**
Erythropoietin stimulating agent	**29 (63.0)**	**25 (36.2)**	**3.00 (1.36–6.62)**		6 (31.6)	5 (10.4)	3.69 (0.95–14.35)		23 (85.2)	20 (95.2)	0.34 (0.03–3.81)		**<0.0001**
Intravenous iron	**26 (56.5)**	**24 (34.8)**	**2.50 (1.14–5.48)**		5 (26.3)	5 (10.4)	3.07 (0.75–12.59)		21 (77.8)	19 (90.5)	0.44 (0.08–2.61)		**<0.0001**
Sevelamer	1 (2.0)	0 (0)	—		0 (0)	0 (0)	—		1 (4.0)	0 (0)	—		
Continuous variables			Partial *R*	*p*			Partial *R*	*p*			Partial *R*	*p*	
Dialysis duration (months)	30 (12–48)	36 (12–48)	−0.090	0.6	—	—	—	—	30 (12–36)	36 (12–42)	−0.138	0.4	—
Phosphate (mmol/l)	1.2 (0.8–1.6)	1.2 (1.0–1.6)	−0.022	0.8	1.3 (0.9–1.6)	1.2 (0.9–1.4)	0.076	0.6	1.3 (0.8–1.7)	1.6 (1.1–2.1)	−0.268	0.07	**0.04**
Calcium (mmol/l)	**2.3 (2.1–2.4)**	**2.3 (2.2–2.4)**	**−0.205**	**0.03**	2.2 (2.1–2.4)	2.3 (2.2–2.4)	−0.120	0.4	2.2 (2.0–2.4)	2.3 (2.2–2.4)	−0.254	0.09	0.2
Calcium × phosphate	2.5 (1.8-3.8)	2.8 (2.2-3.7)	−0.100	0.3	3.0 (2.0–3.6)	2.9 (2.2–3.1)	0.010	0.9	**2.9 (1.8–3.8)**	**3.7 (2.6–4.6)**	**−0.364**	**0.01**	**0.02**
Intact PTH (pg/ml)	**287 (141–531)**	**84 (64–334)**	**0.270**	**0.007**	**232 (92–370)**	**70.0 (50–111)**	**0.426**	**0.001**	369 (172–686)	621 (197–801)	−0.149	0.4	**<0.0001**
Haemoglobin (g/dl)	**10.7 (9.6–12.5)**	**12.6 (10.7–14.2)**	**−0.202**	**0.03**	11.5 (10.0–14.9)	13.5 (10.6–15.1)	−0.072	0.6	10.6 (9.5–11.9)	11.0 (10.7–12.5)	−0.199	0.2	**0.001**
Transferrin saturation (%)	22.0 (17.4–29.0)	22.9 (17.5–30.2)	−0.037	0.7	24.1 (13.7–30.6)	21.0 (17.0–29.0)	−0.144	0.3	22.6 (20.0–30.0)	25.0 (18.5–28.9)	−0.013	0.9	0.3
Ferritin (ng/ml)	**325 (113–573)**	**166 (51–365)**	**0.203**	**0.03**	191 (113–490)	115 (38–224)	0.198	0.1	364 (124–725)	361 (168–609)	0.029	0.8	**0.001**
Albumin (g/l)	36.8 (8.0)	38.6 (5.5)	−0.157	0.1	35.3 (9.2)	38.9 (5.4)	−0.235	0.06	37.8 (7.0)	38.0 (5.9)	−0.025	0.9	0.2
Vitamin D (nmol/l)	19.0 (8.8)	19.8 (9.3)	−0.050	0.6	**15.5 (5.4)**	**20.9 (9.9)**	**−0.269**	**0.03**	21.4 (9.9)	17.4 (7.2)	0.204	0.2	0.07
Uric acid (mmol/l)	0.31 (0.24–0.40)	0.37 (0.28–0.46)	−0.184	0.06	0.38 (0.32–0.48)	0.42 (0.35–50.8)	−0.157	0.2	0.28 (0.21–0.32)	0.28 (0.17–0.34)	0.029	0.9	**<0.0001**
Hs-C-reactive protein (mg/l)	8.2 (2.6–28.1)	6.0 (2.0–15.5)	0.073	0.5	4.7 (1.3–22.3)	4.1 (2.0–15.1)	−0.050	0.7	12.3 (4.1–32.1)	7.7 (1.9–25.4)	0.181	0.2	0.4

Data are expressed as mean (SD), median (interquartile range), or number (percentage). ^a^Adjusted for age and sex; ^b^for differences among black and other African predialysis and black and other African dialysis patients. Significant associations are shown in bold. CV, cardiovascular; ACEI, angiotensin-converting enzyme inhibitor; ARB, angiotensin receptor blocker; OGLA, oral glucose lowering agents; LDL, low-density lipoprotein; HDL, high-density lipoprotein; PTH, intact parathyroid hormone; hs, high sensitivity.

**Table 3:** Arterial function in Black compared to other African chronic kidney disease patients overall and in sensitivity analysis among predialysis and dialysis patients.

Arterial function	Chronic kidney disease patients																		
	All patients						Predialysis patients						Dialysis patients						Intergroup comparison^a^
	Black African (*n* = 46)	Other African (*n* = 69)	Model 1^a^		Model 2^b^		Black African (*n* = 19)	Other African (*n* = 48)	Model 1^a^		Model 2^b^		Black African (*n* = 46)	Other African (*n* = 69)	Model 1^a^		Model 2^b^		*p* value
			Partial *R*	*p*	Partial *R*	*P*			Partial *R*	*p*	Partial *R*	*P*			Partial *R*	*p*	Partial *R*	*P*	
PWV (m/s)	12.0 (3.8)	11.4 (4.4)	0.126	0.2	0.031	0.8	12.2 (3.5)	10.9 (3.7)	0.230	0.09	0.210	0.1	11.9 (4.1)	12.2 (5.5)	0.016	0.9	−0.143	0.4	0.6
Aix (%)	66.6 (16.4)	66.8 (16.7)	0.089	0.4	0.030	0.8	**73.3 (18.0)**	**64.5 (15.7)**	**0.299**	**0.03**	0.229	0.1	62.2 (14.0)	71.8 (18.2)	−0.170	0.3	−0.223	0.2	0.08
RWP (mmHg)	25.0 (13.8–29.0)	20.5 (15.0–25.0)	0.100	0.2	0.152	0.1	**24.0 (14.0–29.0)**	**19.5 (13.5–23.0)**	**0.280**	**0.04**	**0.307**	**0.03**	25.0 (13.0–29.0)	21.5 (15.8–30.5)	0.126	0.4	0.037	0.8	0.1
Rm (%)	67.4 (16.8)	67.2 (16.6)	0.099	0.3	0.047	0.7	**74.3 (18.4)**	**64.9 (15.6)**	**0.309**	**0.02**	**0.250**	**0.09**	62.9 (14.4)	72.3 (18.2)	−0.159	0.3	−0.213	0.2	0.08
CSBP (mmHg)	**136 (22)**	**127 (17)**	**0.265**	**0.006**	**0.225**	**0.02**	**132 (21)**	**125 (15)**	**0.251**	**0.04**	**0.361**	**0.005**	139 (22)	132 (22)	0.208	0.2	0.074	0.7	**0.03**
CPP (mmHg)	**50 (19)**	**43 (13)**	**0.292**	**0.002**	**0.247**	**0.01**	**51 (20)**	**41 (11)**	**0.367**	**0.003**	**0.390**	**0.002**	50 (18)	48 (17)	0.142	0.4	0.056	0.7	**0.04**
PPP (mmHg)	**64 (22)**	**54 (16)**	**0.283**	**0.002**	**0.240**	**0.01**	**65 (23)**	**53 (13)**	**0.358**	**0.003**	**0.405**	**0.001**	64 (22)	59 (21)	0.148	0.3	0.113	0.5	**0.03**
FWP (mmHg)	**35.2 (12.1)**	**31.1 (9.5)**	**0.204**	**0.04**	0.159	0.1	32.5 (12.9)	30.4 (19.2)	0.097	0.5	0.151	0.3	37.0 (11.5)	32.6 (10.2)	0.251	0.1	0.209	0.2	0.1

Descriptive results are expressed as mean (SD) or median (interquartile range). Significant associations are shown in bold. ^a^Adjusted for age and sex; ^b^additionally adjusted for height, weight, heart rate, and mean arterial pressure; ^c^for differences among Black and other African predialysis and Black and other African dialysis patients. PWV, pulse wave velocity; Aix, augmentation index; RWP, reflected wave pressure; Rm, reflection magnitude; CSBP, central systolic blood pressure; CPP, central pulse pressure; PPP, peripheral pulse pressure; FWP, forward wave pressure.

**Table 4:** Left ventricular structure and function in black compared to other African chronic kidney disease patients overall and in sensitivity analysis among predialysis and dialysis patients.

Characteristics	Chronic kidney disease patients												
	All patients				Predialysis patients				Dialysis patients				Intergroup comparison^b^
	Black African (*n* = 46)	Other African (*n* = 69)	Model^a^		Black African (*n* = 19)	Other African (*n* = 48)	Model^a^		Black African (*n* = 27)	Other African (*n* = 21)	Model^a^		*p* value
*Categorical variables*			OR (95% CI)				OR (95% CI)				OR (95% CI)		
LV hypertrophy	21 (45.7)	27 (39.1)	1.50 (0.65 to 3.48)		6 (31.6)	18 (37.5)	0.77 (0.22 to 2.75)		15 (55.6)	9 (42.9)	1.78 (0.55 to 6.27)		0.3
LV concentric remodelling	15 (33.3)	18 (26.9)	1.36 (0.59 to 3.15)		7 (36.8)	13 (27.7)	1.53 (0.49 to 4.78)		8 (30.8)	5 (25.0)	1.35 (0.35 to 5.14)		0.9
Reduced ejection fraction	5 (11.1)	17 (25.3)	0.41 (0.14 to 1.26)		2 (10.5)	11 (23.9)	0.46 (0.07 to 2.62)		3 (11.5)	6 (30.0)	0.32 (0.07 to 1.52)		0.3

*Continuous variables*			Partial *R*	*p*			Partial *R*	*p*			Partial *R*	*p*	
LV mass index (g/m^2^)	104.1 (50.9)	91.4 (38.6)	0.147	0.1	99.2 (38.2)	88.6 (38.2)	0.132	0.3	107.6 (58.8)	97.9 (39.6)	0.084	0.06	0.3
LV relative wall thickness	0.39 (0.12)	0.38 (0.12)	0.023	0.8	0.42 (0.13)	0.38 (0.10)	0.132	0.3	0.37 (0.10)	0.39 (0.15)	−0.058	0.7	0.6
Ejection fraction (%)	65.9 (13.7)	61.2 (14.6)	0.159	0.1	65.7 (15.7)	63.3 (13.8)	0.071	0.6	**66.1 (12.3)**	**56.4 (15.8)**	**0.356**	**0.01**	0.1
Stroke volume (ml/beat)	74.1 (24.7)	66.9 (24.2)	0.150	0.1	67.6 (22.3)	69.2 (24.3)	−0.025	0.9	**78.9 (25.7)**	**61.6 (23.6)**	**0.315**	**0.03**	0.1
*E*/*A*	1.10 (0.48)	1.01 (0.36)	0.053	0.6	1.19 (0.56)	1.06 (0.38)	0.093	0.5	1.04 (0.41)	0.91 (0.27)	0.135	0.4	0.2
*E*/*e*′	**11.5 (5.1)**	**9.7 (4.0)**	**0.254**	**0.007**	**11.3 (5.4)**	**8.8 (3.6)**	**0.307**	**0.01**	11.7 (4.1)	11.7 (5.0)	0.070	0.7	**0.02**
Average *e*′ (cm/s)	**8.2 (2.6)**	**8.7 (2.7)**	**−0.201**	**0.03**	**8.4 (2.4)**	**9.1 (2.7)**	**0.246**	**0.04**	8.0 (2.8)	7.8 (2.6)	−0.049	0.8	0.2

Data are expressed as mean (SD), median (interquartile range), or number (percentage). ^a^Adjusted for age and sex; ^b^for differences among black and other African predialysis and black and other African dialysis patients. Significant associations are shown in bold. LV, left ventricular; *E*, early diastolic wave; *A*, late or atrial diastolic wave; *e*′, peak velocity during early diastole.

**Table 5:** Carotid atherosclerosis and cardiovascular event rates in black compared to other African chronic kidney disease patients overall and in sensitivity analysis among predialysis and dialysis patients.

Characteristic	Chronic kidney disease patients												
	All patients				Predialysis patients				Dialysis patients				Intergroup comparison^b^
	Black African (*n* = 46)	Other African (*n* = 69)	Model^a^		Black African (*n* = 19)	Other African (*n* = 48)	Model^a^		Black African (*n* = 27)	Other African (*n* = 21)	Model^a^		*p* value
*Categorical variables*			OR (95% CI)				OR (95% CI)				OR (95% CI)		
Carotid atherosclerosis													
Plaque	**14 (30.4)**	**40 (58.8)**	**0.38 (0.16–0.91)**		7 (36.8)	29 (61.7)	0.43 (0.12–1.51)		7 (25.9)	11 (52.4)	0.35 (0.10–1.29)		**0.01**
Cardiovascular events													
Heart failure	4 (8.7)	8 (11.6)	0.79 (0.22–2.86)		1 (5.3)	4 (8.3)	0.68 (0.07–6.72)		3 (11.1)	4 (19.1)	0.59 (0.11–3.08)		0.5
Ischemic heart disease	4 (8.7)	18 (26.1)	0.33 (0.10–1.15)		4 (21.1)	11 (22.9)	1.26 (0.31–5.23)		0 (0)	7 (33.3)	—		**0.02**
Peripheral arterial disease	0 (0)	2 (2.9)	—		0 (0)	2 (4.2)	—		0 (0)	0 (0)	—		—
Any cardiovascular event	8 (17.4)	24 (34.8)	0.49 (0.19–1.30)		4 (21.1)	14 (29.2)	0.93 (0.22–3.87)		**4 (14.8)**	**10 (47.6)**	**0.22 (0.05–0.88)**		0.07
*Continuous variable*			Partial *R*	*p*			Partial *R*	*p*			Partial *R*	*p*	
Carotid atherosclerosis													
Intima-media thickness (mm)	0.610 (0.580–0.725)	0.690 (0.560–0.875)	−0.116	0.2	0.650 (0.580–0.725)	0.690 (0.560–0.875)	−0.058	0.6	0.580 (0.503–0.691)	0.645 (0.545–0.763)	−0.095	0.5	**0.04**

Data are expressed as number (percentage) or median (interquartile range) as appropriate. ^a^Adjusted for age and sex; ^b^for differences among black and other African predialysis and black and other African dialysis patients. Significant associations are shown in bold.

**Table 6:** Cardiovascular risk factors in black and other African chronic kidney disease patients with and without carotid plaque.

CVD risk factor	Chronic kidney disease patients					
	Black African patients			Other African patient		
	Plaque (*n* = 14)	No plaque (*n* = 32)	OR (95% CI)	Plaque (*n* = 40)	No plaque (*n* = 28)	OR (95% CI)
Age (years)	57.6 (15.9)	52.8 (14.0)	1.02 (0.98 to 1.07)	**66.1 (8.3)**	**52.1 (14.0)**	**1.13 (1.06 to 1.20)**
Female sex	7 (50)	11 (34.4)	1.90 (0.53 to 6.84)	**7 (17.5)**	**18 (64.3)**	**0.12 (0.04 to 0.36)**
Hypertension	14 (100)	31 (96.8)	—	34 (85.0)	25 (85.7)	0.94 (0.24 to 3.71)
Dyslipidemia	11 (78.6)	19 (59.4)	2.90 (0.53 to 15.69)	**33 (82.5)**	**19 (67.9)**	**13.90 (1.61 to 119.78)**
Smoking	0 (0)	2 (6.3)	—	1 (2.5)	0 (0)	—
Framingham score	25.0 (20.5)	22.7 (22.4)	1.01 (0.98 to 1.03)	**29.8 (15.8)**	**13.2 (11.7)**	**1.10 (1.04 to 1.16)**

Data are expressed as mean (SD) or number (percent) and were analysed in logistic regression models. Significant associations are shown in bold. CVD, cardiovascular disease.

## Data Availability

The data used in this study can be obtained from the corresponding author.

## Supplementary Materials

Supplementary Materials Methods used in cardiovascular risk factor and arterial and left ventricular systolic and diastolic function recording.
