# Supplementary material for: Cardiovascular Risk Factor Profiles and Disease in Black Compared to Other Africans with Chronic Kidney Disease
Source: Int J Nephrol. 2021 Feb 19;2021:8876363. doi: 10.1155/2021/8876363 (PMC7929676; doi:10.1155/2021/8876363)
Supplement: Supplementary Materials — Methods used in cardiovascular risk factor and arterial and left ventricular systolic and diastolic function recording. [file 8876363.f1.docx]

**Supplementary material**

**Methods**

*Cardiovascular risk factors.* Hypertension was identified when the systolic or/and diastolic blood pressure were ≥ 140mmHg and 90mmHg, respectively, or/and antihypertensive agents were used. Analyses were performed on fasting blood samples using routine laboratory tests for the determination of lipid, glucose, phosphate, calcium, haemoglobin, albumin, uric acid and ferritin concentrations, transferrin saturation percentages. Intact parathyroid hormone levels were measured by an electrochemiluminescent immunoassay on Roche Cobas. High-sensitive C-reactive protein and vitamin D concentrations were both estimated on Abbott Architect using an immunoturbidimetric assay and chemoluminescent microparticle immunoassay, respectively. Dyslipidemia was diagnosed when the total cholesterol: HDL cholesterol ratio was >4 or/and lipid lowering drugs were employed. Diabetes was identified in patients with a fasting glucose level of >7 mmol/l and in those that used glucose lowering medications.

*Arterial function.* Subsequent to resting for 15 minutes in the supine position, arterial waveforms at the radial (dominant arm), carotid and femoral artery pulses were recorded for a time period of ten consecutive waveforms (heart beats). Calibration of the pulse wave was done by manual measurement (auscultation) of brachial BP taken immediately prior to recordings. A validated generalized transfer function incorporated in the SphygmoCor software was used to convert the peripheral pressure wave form into a central aortic waveform. The results were discarded when the systolic or diastolic variability of consecutive waveforms exceeded 5% or the amplitude of the pulse wave signal was less that 80 mV. The aortic pulse wave velocity was calculated as distance in meters divided by transit time in seconds. The time delay in pulse waves between the carotid and femoral sites was assessed by employing an ECG-derived R wave as a fudicial point. Pulse transit time was calculated as the mean of 10 consecutive beats. The difference between the distance from the femoral sampling site to the suprasternal notch and the distance from the carotid sampling site to the suprasternal notch represented the distance the pulse wave travels. Magnitude of the forward and reflected wave components of the aortic pressure waveform was determined by the SphygmoCor software, which separates the aortic waveform by using a modified triangular waveform. Augmentation index was calculated as (second systolic peak/first systolic peak) x 100, reflection magnitude as (reflected wave amplitude/forward wave amplitude) x 100 and pulse pressure amplification as (radial pulse pressure/aortic pulse pressure) x 100. All measurements were made by a single experienced observer(CR) who was unaware of the cardiovascular risk factor profiles of the patients. Brachial blood pressure was recorded in all patients. Technically sound measurements of the central pressure wave and pulse wave velocity were obtained in 151 and 140 patients, respectively.

*Left ventricular structure and function.*Echocardiography was performed with the patient in the partial left decubitus position.Left ventricular dimensions were determined by measuring the left ventricular internal end diastolic and end systolic diameters and wall thickness (inter-ventricular septal and posterior wall thickness) in the parasternal long axis view by two-dimensional directed M-mode echocardiography. Left ventricular end diastolic and systolic volumes were assessed using the Teichholz method[1]. Stroke volume was determined from the difference between LV end diastolic and systolic volumes as evaluated upon employing the Z-derived method [2].Left ventricular ejection fraction was calculated as [(left ventricular end diastolic volume –left ventricular end systolic volume) / left ventricular end diastolic volume] x 100. Left ventricular mass was determined using a standard formula [3] and indexed to body surface area(left ventricular mass index). Left ventricular relative wall thickness was calculated as (left ventricular diastolic posterior wall thickness x 2) / left ventricular end diastolic diameter [4]. Left ventricular hypertrophy was identified when left ventricular mass index was >95 g/m^2^ for women and >115 g/m^2^ for men [3].

Left ventricular diastolic function was assessed using pulsed Doppler and tissue Doppler imaging [5]. Transmitral flow patterns were recorded at the mitral valve leaflet tips using pulsed Doppler in the apical four chamber view. From the mitral valve inflow velocity curve, the early (E) and late (atrial-A) diastolic wave were measured and the ratio of early and late diastolic filling (E/A) was calculated. To perform tissue Doppler imaging, the velocity of myocardial tissue lengthening was measured by placing the curser at the septal and lateral corners of the mitral annulus in the apical four chamber view. To determine diastolic function using tissue Doppler imaging, the early diastolic mitral annulus motion (e’) was recorded. Because mitral E is dependent on ventricular relaxation as well as left atrial driving forces (pressures), while e’ is dependent on relaxation alone, E/e’ ratio is considered to be an index of left ventricular filling pressures. The E/e’ ratio was calculated as mitral E/the average of septal and lateral e’. Echocardiographic measurements were made by the same observer that performed the arterial function evaluation (CR). Intra-observer variation for echocardiographic measurements is low in our setting [6].

**References**

[1] L.E. Teichholz, T. Kreulen, M.V. Herman, R. Gorlin, “Problems in echocardiographic volume determinations: echocardiographic-angiographic correlations in the presence or absence of asynergy,” *Am J Cardiol*, vol. 37, no. 7, pp. 7-11, 1976.

[2] G. De Simone, R.B. Devereux, A. Ganau et al.,“Estimation of the left ventricular chamber and stroke volume by limited M-Mode echocardiography and validation by two-dimensional and Doppler echocardiography,”*Am J Cardiol*, vol. 78, no. 7, pp. 801-807, 1996.

[3] R.M. Lang, L.P. Badano, V. Mor-Avi et al.,“Recommendations for cardiac chamber quantification by echocardiography in adults: an update from the American Society of Echocardiography and the European Association of Cardiovascular Imaging,” *Eur Heart J Cardiovasc Imaging*, vol. 16, no. 3, pp. 233-271, 2015.

[4] A. Ganau, R.B. Devereux, M.J. Roman et al.,“Patterns of left ventricular hypertrophy and geometric remodeling in essential hypertension,”*J Am Coll Cardiol*, vol. 19, no. 7, pp. 1550-1558, 1992.

[5] S.F. Nagueh, O.A. Smiseth, C.P. Appleton et al.,“Recommendations for the evaluation of left ventricular diastolic function by echocardiography: An update form the American Society of Echocardiography and the European Association of Cardiovascular Imaging,”*J Am Soc Echocardiogr,* vol. 29, no. 4, pp. 277-314, 2016.

[6] L. Mokotedi, S. Gunter, C. Robinson et al.,“[The Impact of different classification criteria sets on the estimated prevalence and associated risk factors of diastolic dysfunction in rheumatoid arthritis,”](https://www.ncbi.nlm.nih.gov/pubmed/29348754) *Int J Rheumatol* 2017;2017:2323410.
